# Bias in AI-based models for medical applications: challenges and mitigation strategies

**DOI:** 10.1038/s41746-023-00858-z

**Published:** 2023-06-14

**Authors:** Mirja Mittermaier, Marium M. Raza, Joseph C. Kvedar

**Affiliations:** 1 Charité—Universitätsmedizin Berlin, Corporate Member of Freie Universität Berlin and Humboldt-Universität zu Berlin, Department of Infectious Diseases, Respiratory Medicine and Critical Care, Berlin, Germany; 2 Berlin Institute of Health at Charité—Universitätsmedizin Berlin, Charitéplatz 1, 10117 Berlin, Germany; 3 Harvard Medical School, Boston, MA USA

**Keywords:** Health policy, Computational models

## Abstract

Artificial intelligence systems are increasingly being applied to healthcare. In surgery, AI applications hold promise as tools to predict surgical outcomes, assess technical skills, or guide surgeons intraoperatively via computer vision. On the other hand, AI systems can also suffer from bias, compounding existing inequities in socioeconomic status, race, ethnicity, religion, gender, disability, or sexual orientation. Bias particularly impacts disadvantaged populations, which can be subject to algorithmic predictions that are less accurate or underestimate the need for care. Thus, strategies for detecting and mitigating bias are pivotal for creating AI technology that is generalizable and fair. Here, we discuss a recent study that developed a new strategy to mitigate bias in surgical AI systems.

## Bias in medical AI algorithms

Artificial intelligence (AI) technology is increasingly applied to healthcare, from AI-augmented clinical research to algorithms for image analysis or disease prediction. Specifically, within the field of surgery, AI applications hold promise as tools to predict surgical outcomes^1^, aid surgeons via computer vision for intraoperative surgical navigation^2^, and even as algorithms to assess technical skills and surgical performance^1,3–5^.

Kiyasseh et al.^4^ highlight this potential application in their work deploying surgical AI systems (SAIS) on videos of robotic surgeries from three hospitals. They used SAIS to assess the skill level of surgeons completing multiple different surgical activities, including needle handling and needle driving. In applying this AI model, Kiyasseh et al.^4^ found that it could reliably assess surgical performance but exhibited bias. The SAIS model showed an underskilling or overskilling bias at different rates across surgeon sub-cohort. Underskilling was the AI model downgrading surgical performance erroneously, predicting a particular skill to be lower quality than it actually was. Overskilling was the reverse—the AI model upgraded surgical performance erroneously, predicting a specific skill to be of higher quality than it was. Underskilling and overskilling were measured based on the AI-based predictions’ negative and positive predictive values negative, respectively.

## Strategies to mitigate bias

The issue of bias being exhibited, perpetuated, or even amplified by AI algorithms is an increasing concern within healthcare. Bias is usually defined as a difference in performance between subgroups for a predictive task^6,7^. For example, an AI algorithm used for predicting future risk of breast cancer may suffer from a performance gap wherein black patients are more likely to be assigned as “low risk” incorrectly. Further, an algorithm trained on hospital data from German patients might not perform well in the USA, as patient population, treatment strategies or medications might differ. Similar cases have already been seen in healthcare systems^8^. There could be many different reasons for this performance gap. Bias can be generated across AI model development steps, including data collection/preparation, model development, model evaluation, and deployment in clinical settings^9^. With this particular example, the algorithm may have been trained on data predominantly from white patients, or health records from Black patients may be less accessible. Additionally, there are likely underlying social inequalities in healthcare access and expenditures that impact how a model might be trained to predict risk^6,10^. Regardless of the cause, the impact of an algorithm disproportionately assigning false negatives would include fewer follow-up scans, and potentially more undiagnosed/untreated cancer cases, worsening health inequity for an already disadvantaged population. Thus, strategies to detect and mitigate bias will be pivotal to improving healthcare outcomes. Bias mitigation strategies may involve interventions such as pre-processing data through sampling before a model is built, in-processing by implementing mathematical approaches to incentivize a model to learn balanced predictions, and post-processing^11^. Further, as experts can be aware of biases specific to datasets, “keeping the human in the loop” can be another important strategy to mitigate bias.

With their SAIS model, Kiyasseh et al.^4^ developed a strategy called TWIX to mitigate bias. TWIX is an add-on application that taught the SAIS model to add a prediction of the importance of video clips that was used to assess surgical skill. They hypothesized that the SAIS model’s bias might be due to the system latching onto unreliable video frames for assessment. TWIX requiring model predictions of video clip importance served a similar role to human assessors explaining the rationale for assessments. Kiyasseh et al.^4^ found that TWIX mitigated SAIS model bias, improving model performance both for the disadvantaged surgeon sub-cohorts and for surgical skill assessments overall. This accomplishment is beneficial not only for this particular use case but also implies that this type of bias mitigation strategy could be used to continue to improve AI applications in the future.

## A look into the future—challenges with continuously learning AI models

Bias within AI algorithms must continue to be studied and mitigated as AI technology develops. Looking into the future, one question that will most definitely arise is what level of bias is acceptable for an AI algorithm^4^. This is analogous to the question of what accuracy threshold is acceptable for a particular AI system^4^. Previous groups suggested that any performance discrepancy is indicative of algorithmic bias, but expecting completely bias-free systems before implementation is unrealistic^12^. Performance discrepancy may also differ based on the data and population an AI algorithm is trained on and then subsequently applied to. Currently, there is significant heterogeneity in terms of the datasets AI algorithms are trained with within algorithm types themselves^13,14^. The question of whether AI algorithms may need to be more generalizable, trained on larger and more diverse datasets to be applied to broader populations, or more localized and applied narrowly remains to be addressed. In any case, AI models will have to be explainable^15^ with transparent methodologies so that these questions can be studied and debated in the coming years.

Another issue for the future is whether AI algorithms will be able to be changed/edited, just as Kiyasseh et al.^4^ added TWIX to their existing SAIS algorithm. An AI algorithm can either be locked—once the algorithm is trained, the model provides the same result when the same input is applied—or adaptive^16^. In this case, the AI model could be updated continuously as it learns from new data over time rather than becoming outdated within a few years. However, continuous learning also possesses the risk of increasing or adding new bias if the new data are biased^17^. Thus, methodologies for regular bias detection and continual bias mitigation will be key to AI implementation.

From a regulatory standpoint, new initiatives also aim to tackle the issue of biased data in AI systems. The STANDING Together initiative (standards for data diversity, inclusivity, and generalizability), launched in September 2022, aims to develop recommendations for the composition (who is represented) and reporting (how they are represented) of datasets underpinning medical AI systems^18^. Further, the FDA has recognized challenges due to bias in AI and ML algorithms and released an action plan (“Artificial Intelligence/Machine Learning (AI/ML)-Based Software as a Medical Device (SaMD) Action Plan”) in January 2021^9,19^, emphasizing the importance of identifying and mitigating bias in AI systems^9^. As part of the FDA Action Plan, the FDA intends to support the piloting of real-world performance monitoring^19^, allowing for the detection of bias after deployment. Further, to meet regulatory challenges that come with continuously adopting AI models, the FDA recently released a draft guidance to develop a less burdensome regulatory approach supporting the iterative improvement of, e.g., AI models while continuing to assure their safety and effectiveness^20^. These types of regulatory steps should be encouraged, as they will become increasingly necessary to ensure the minimization of bias without the blockade of AI innovation.

## Conclusion

The integration of AI into medical technology and healthcare systems is only going to increase in the coming years. Key to AI model integration and usability will be bias mitigation. Kiyasseh et al. describe an innovative approach to bias mitigation with their TWIX system. As technology continues to develop, the push toward bias mitigation occurs at all levels—from model development and over training to deployment and implementation. This effort will require checks and balances from innovators, healthcare institutions, and regulatory entities.

### Reporting summary

Further information on research design is available in the Nature Research Reporting Summary linked to this article.

## Supplementary information

Reporting Summary
